# Outcomes of Implementing in the Real World the Women's Health CoOp Intervention in Cape Town, South Africa

**DOI:** 10.1007/s10461-021-03251-7

**Published:** 2021-04-23

**Authors:** Wendee M. Wechsberg, Felicia A. Browne, Jacqueline Ndirangu, Courtney Peasant Bonner, Tracy L. Kline, Margaret Gichane, William A. Zule

**Affiliations:** 1 Substance Use, Gender, and Applied Research Program, RTI International, 3040 E. Cornwallis Road, Research Triangle Park, NC 27709-2194 USA; 2 Maternal and Child Health, University of North Carolina Gillings School of Global Public Health, 135 Dauer Drive, Chapel Hill, NC 27599 USA; 3 Department of Psychology, North Carolina State University, 640 Poe Hall, Campus, Box 7650, Raleigh, NC 27695 USA; 4 Psychiatry and Behavioral Sciences, Duke University School of Medicine, 40 Duke Medicine Circle, Durham, NC 27710 USA; 5 Health Behavior, University of North Carolina Gillings School of Global Public Health, 135 Dauer Drive, Chapel Hill, NC 27599 USA; 6 Division for Statistical and Data Sciences, RTI International, 3040 E. Cornwallis Road, Research Triangle Park, NC 27709 USA

**Keywords:** Implementation science, Gender-focused, HIV intervention, ART adherence, Patient satisfaction, Hybrid design

## Abstract

Women in South Africa living with HIV who use alcohol may not adhere to ART, affecting the country’s 90-90-90 targets. The Women’s Health CoOp (WHC), a woman-focused HIV intervention, has shown efficacy in numerous trials with key populations of women in South Africa who use alcohol and drugs. In a hybrid implementation effectiveness study, the WHC was implemented in usual care clinics by healthcare providers in a modified stepped-wedge design. We present the outcomes of alcohol use and ART adherence with 480 women, with a 95% 6-month follow-up rate across 4 implementation cycles. Compared with the first cycle, women in the fourth cycle were significantly less likely (OR = 0.10 [95% CI 0.04, 0.24]) to report alcohol use disorder risk and were 4 times more likely (OR = 4.16 [95% CI 1.05, 16.51]) to report ART adherence at 6-month follow-up. Overall, acceptability and satisfaction were extremely high. The WHC intervention was successful in reaching key populations of women to reduce alcohol use and increase ART adherence, which is essential for South Africa to reach the 90-90-90 goals.

## Introduction

In South Africa, alcohol use is a major public health concern that intersects with high HIV rates among women of reproductive age, with approximately 17% of women reporting hazardous or harmful drinking (i.e., based on the Alcohol Use Disorders Identification Test (AUDIT)) [1–3]. Additionally, women in South Africa experience a disproportionate burden of the HIV epidemic—the latest national surveillance estimates indicate the highest HIV prevalence is among people aged 25 to 49, where it is significantly higher among women (33.3%) as compared with men (19.9%) [4].

Within the HIV treatment cascade, an estimated 64% of women living with HIV are on antiretroviral therapy (ART) and among these women, 58% are virally suppressed [4]. While progress has been made on ART initiation and adherence, these numbers are short of the UNAIDS 95-95-95 treatment targets for 2030, much less the previous 90-90-90 treatment targets for 2020 [5]. Heavy alcohol use among people living with HIV is prevalent and may impede progress toward these goals. Among 354 South African women living with HIV, 85% of women reported engaging in heavy drinking (i.e., 4 or more drinks on any given day) in the past month [6] (W. Wechsberg, unpublished data, 2018). Alcohol use is associated with reduced ART adherence and healthcare utilization [7]; increased risk of comorbid conditions and disease progression [8]; and sexual risk behavior, which can lead to onward transmission of HIV [9, 10]. Further, South Africa has the highest prevalence globally of fetal alcohol spectrum disorders caused by alcohol use during pregnancy [11, 12], in addition to countrywide gender-based violence [13, 14].

Access to substance use treatment is limited in South Africa, especially for women living in economically disadvantaged communities [15, 16]. In the Western Cape of South Africa, women make up 27% of admissions to substance use treatment facilities. Structural barriers, such as long travel time to substance use treatment facilities and competing financial responsibilities, disproportionately restrict women from accessing treatment compared with men, in addition to the lack of women-centered treatment [17]. These barriers are compounded by stigma related to people with substance use problems and negative perceptions about the efficacy of substance use treatment programs [18]. Similarly, barriers to HIV treatment include financial barriers—such as the cost of transportation—and a lack of proper knowledge about HIV [19, 20]. Given these significant overlapping barriers, women living with HIV who use alcohol are a highly vulnerable population in need of targeted interventions to get them into care and ultimately to be virally suppressed.

Efficacious interventions that address the intersection of HIV and alcohol use may help improve HIV treatment adherence [21, 22]. The Women’s Health CoOp (WHC), a brief, evidence-based, woman-focused, behavioral intervention grounded in empowerment and feminist theory, is one such intervention. The WHC uses a skill-building approach with the aim of reducing various HIV-related risks for populations of women who use alcohol and other drugs, specifically addressing the intersection of alcohol and other drug use, sexual risk behavior, and gender-based violence. Further, it works to promote initiation of and adherence to biomedical HIV treatment and prevention strategies with more recent versions including ART and pre-exposure prophylaxis (PrEP) [1, 23].

This intervention was developed in the US for African American women who use substances, and the original Women’s CoOp was found to be a top-tier best evidence HIV risk-reduction intervention by the Centers for Disease Control and Prevention review of Best Evidence interventions [24]. The WHC has been adapted to various key populations and settings in South Africa and has been found to be efficacious in reducing risks for women who use alcohol and other drugs [1, 2, 25]. Two key populations in South Africa are high-risk drinkers (i.e., elevated AUDIT scores) and recreational drug users as they have had higher HIV prevalence than the general population [3, 4, 26]. In South Africa, the WHC adaptation that reaches these key populations was listed in USAID’s “Integrating Multiple Gender Strategies to Improve HIV and AIDS Interventions: A Compendium of Programs in Africa” [27]. More recently, the adaptation incorporated biological approaches/interventions into the behavioral intervention, such as ART [1]. In a recent cluster randomized trial of the WHC with biobehavioral approaches, women in the WHC arm were less likely to report heavy drinking (i.e., OR = 0.45); physical abuse (i.e., OR = 0.41), and sexual abuse (i.e., OR = 0.40). Those in the WHC arm also were more likely to report condom use with boyfriends (i.e., OR = 1.63). Additionally, reductions in viral loads were observed for a subsample of WHC participants [1].

The original intervention and its adaptations have been implemented in randomized field experiments, with trained research project staff from the local communities delivering the intervention for almost 20 years in South Africa and have demonstrated efficacy [1, 2, 25, 28, 29]. Several randomized trials of the WHC in South Africa have shown that the intervention is associated with reductions in alcohol use and sexual risk behavior among key populations of women [2, 25, 29]. However, to achieve population-level changes in these risk behaviors, the next step is to shift from randomized trials to implementation studies to understand how evidence-based interventions are implemented in the real world.

Most successful interventions take an average of 17 years to translate research into practice [30, 31]. Consequently, the goal was to translate and disseminate the WHC for greater reach by implementing the WHC into usual care settings and assessing its success with women who are most at risk for or living with HIV. Hybrid implementation science designs offer methods of translation into diverse settings to demonstrate evidence not only of practice and policy [32, 33] but also patient effectiveness [34]. Hybrid designs in the real world, especially within economically disadvantaged communities where local health clinics and substance use treatment centers reside and reach key populations, are essential to determine true effectiveness.

This article presents the patient outcomes from an implementation science trial using a modified stepped-wedge study design of the WHC in public health clinics and substance use treatment facilities in Cape Town, South Africa. Specifically, it examines the impact of the intervention on the patient-level outcomes of risk for alcohol use disorder and ART adherence. The appropriateness, acceptability, and feasibility of the WHC in usual care settings have been reported previously [35].

## Methods

The implementation science trial began in September 2015 and ended in December 2018. Study approvals were granted by the South African Medical Association Research Ethics Committee (SAMAREC); the City of Cape Town: City Health Research Committee; and the RTI International Office of Research Protection Institutional Review Board. This study was registered in Clinical Trials (ClinicalTrials.gov Identifier: NCT02733003). Information about the study protocol has been reported previously [36].

### Study Design

**Fig. 1:**
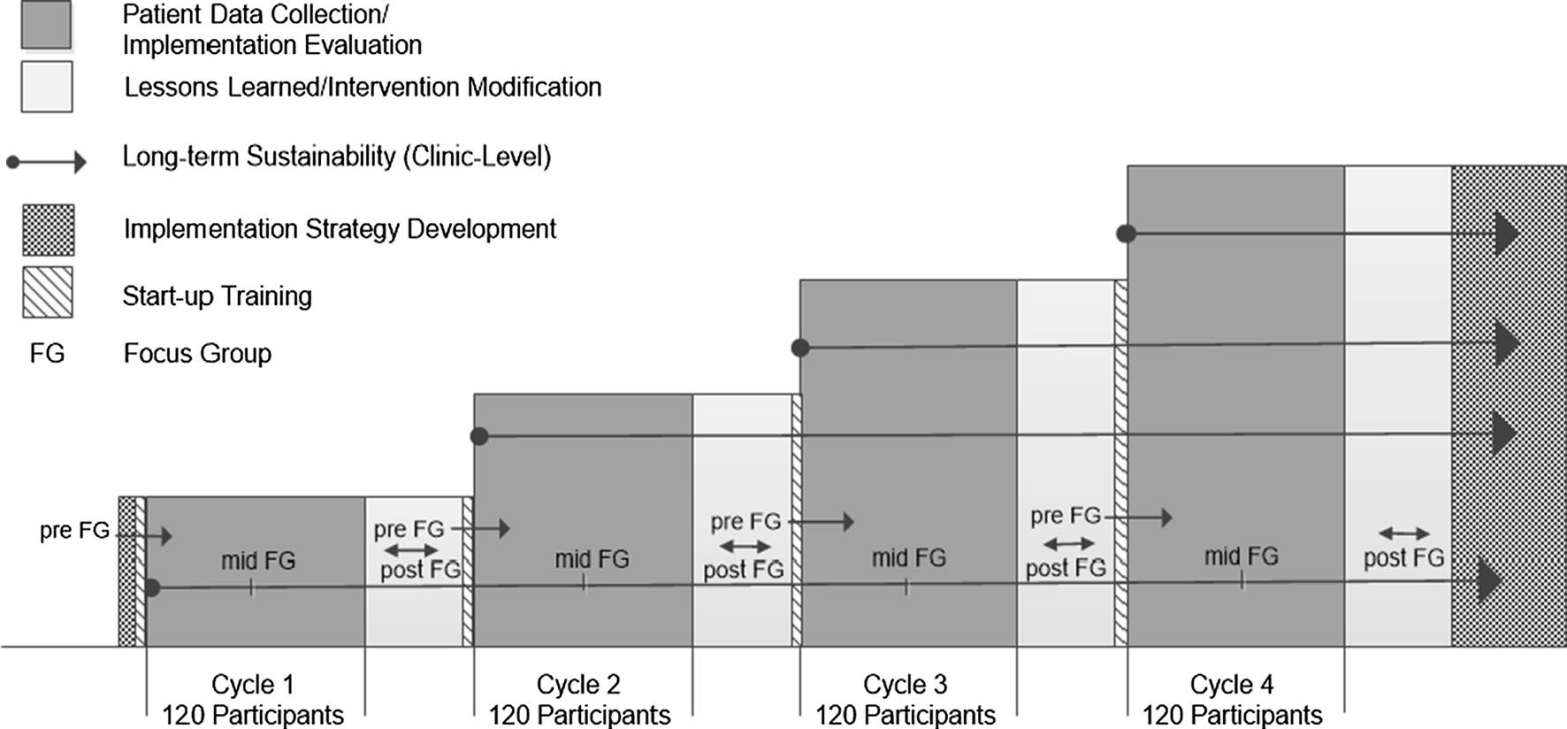
Modified study design

This study used a modified cluster-randomized stepped-wedge design. In a cluster-randomized stepped-wedge design, each cluster receives the intervention; however the order in which clusters receive the intervention is randomized. This approach overcomes the ethical issues that would arise from a traditional cluster-randomized trial in which participants in clusters assigned to the control arm would not receive an intervention that had already been proven to be efficacious. It also overcomes logistical issues associated with trying to implement an intervention in a large number of clusters simultaneously. These features make the stepped-wedge design particularly well suited for studies assessing the implementation of interventions with established efficacy. A more simplified study design is shown in Fig. 1.

### Clinic Inclusion and Randomization

In this study, clusters comprised eight clinics—4 HIV/antenatal clinics and 4 substance use treatment rehab clinics—in Cape Town, selected and run by the City of Cape Town Health Department. The clinics were each embedded within clinic compounds that served populations of African descent in historically underserved communities where there is a higher prevalence of HIV. All clinics received the intervention. Randomization was conducted using a SAS computer program to assign clinic pairs matched by geography—one HIV clinic and one substance use treatment clinic—to 1 of 4 cycles. The implementation strategies and the WHC intervention were conducted one cycle at a time—with Cycle 1 occurring for the first 6 months, and each subsequent cycle following until Cycle 4 was complete. Prior to each cycle, the study’s Principal Investigator trained clinic staff at both clinics to administer the WHC intervention.

### Patient Screening and Inclusion

This modified stepped-wedge design recruited 480 women living with HIV. Project staff conducted outreach activities in the communities around the study clinics and in-reach activities within the clinics. Project staff asked interested individuals a set of eligibility questions using a screener. To be eligible, potential participants had to meet the following criteria: be female; be 18 to 45 years of age; report the use of at least one substance—which could include alcohol—at least weekly during the previous 3 months; report unprotected sex (sex without a condom) with a male partner in the past 6 months; had a positive HIV test result from either the participating health clinic or treatment clinic, or a clinic-issued ART appointment card or ART prescription as verification of HIV status; and intend to remain in the area for at least 6 months. Individuals were excluded if they were not HIV-positive or if they were unwilling to participate in alcohol and other drug screening tests. Individuals who met eligibility criteria and were interested in participating were scheduled for an intake appointment (if not the same day) at one of the clinics, where they were rescreened for eligibility and then enrolled if they provided informed consent to participate.

### Intake Process

If the participant could not attend the baseline appointment on the same day of her screening, she was given a date of her choice for the appointment where she was rescreened for eligibility using the same screening form used previously to verify responses. The intake appointment was conducted in the study clinic. Research staff obtained informed written and signed consent and a signed release for referral for health services and administered a face-to-face baseline interview using a secure tablet with skip patterns and quality-control checks programmed into the interview. The baseline interview included measures of socioeconomic status; sex risk behaviors; alcohol and other drug use; substance use treatment readiness; ART use and barriers to use; and other self-reported clinical outcomes, such as tuberculosis (TB) and symptoms of sexually transmitted infections (STIs). Biological data were obtained from a urine specimen tested for pregnancy and metabolites for recent drug use. Participants also underwent a breathalyzer test for recent alcohol use. Active linkage to ART initiation, antenatal care, and substance use treatment was offered at the end of the appointment. Participants were given R100 for their time and transportation reimbursement. During this appointment, they also were scheduled for their first WHC intervention workshop.

### Women’s Health CoOp (WHC) Intervention

A master trainer trained clinic staff after the initial training with the PI at each study clinic on the intervention two weeks prior to implementation launch to practice and role-play competence [23]. These trained clinic staff led the WHC intervention—a 2-session group workshop—at the participating study clinics. Clinic staff included HIV counseling and testing staff, nurses, clerks, and paraprofessionals. Workshop groups averaged in size with 5 attendees—and participants could attend the workshop in either order for flexibility of the workshop and for real-world implementation. Workshop 1 content consisted of facts about HIV, STIs, and TB; male and female condoms; sex risk reductions; skills to negotiate safer sex; and how women living with HIV can stay healthy. Workshop 2 content consisted of facts about alcohol and other drugs, injection drugs, reducing alcohol and other drug risk, violence, and prevention strategies. An individual personalized action plan was developed, when possible, at the end of the second workshop. Each workshop lasted an average of 46 min (depending on the discussion) and the two workshops usually took a total of less than two hours together and both workshops were held approximately within a month, although this time varied depending on when enough participants were available to convene a workshop group. An overview of the intervention content and topics for each workshop is presented in Table 1.

**Table 1:** Overview of Women’s Health CoOp intervention, by workshop title slides

Workshop 1—Titles of slides and some associated content on the intersection with alcohol and drug use
Why reach women? Unprotected sex, using drugs & drinking alcohol before sex, lack of condoms, violence associated with alcohol and drug use
Other reasons women are at risk in South Africa: lack of power, skills, high rates of abuse, lack of choice
How do women get infected with HIV? Women who use alcohol or drugs may forget to use a condom, other partner risk
What we want you to know about HIV
Other facts you need to know about HIV
Over time, signs and symptoms of HIV begin, if not on medication
Concerns about telling others of HIV test results
Meaning of an HIV-positive test result
CD4 and viral load tests
ARV treatment and viral load
Importance of good adherence
Adherence tips
HIV and TB
Risks of smoking among HIV-positive women
What about breastfeeding?
Reducing the risk of unplanned pregnancy
STI symptoms women should know
Other risks
Do you know what STIs look like?
Male and female extreme HPV (genital warts)
Male and female herpes
The male condom
Sexy safer sex and pleasure
How to use a male condom
Male circumcision decreases HIV risk for men
Women’s sex organs are more hidden than men’s
You must keep your private parts (vagina) healthy
About the female condom: there are two kinds
Steps on how to use a female condom
Activity: practise
Oral protection for women
How to talk with your partner about safer sex
Tips for effective refusal
Negotiate for sexy safer sex
Improving & protecting my health

Workshop 2—Titles of slides and some associated content on the intersection with alcohol and drug use
Alcohol and drug abuse in South Africa
Alcohol and other drug (AOD) use compromises behaviour (AOD > Risky Sex > Violence > STIs/HIV)
Why is alcohol so risky for women?
Alcohol and drugs affect unborn babies
Risks of tobacco use
Facts about “Dagga”: weed, marijuana
Facts about mandrax “Buttons”
Facts about “TIK”—Methamphetamine (“Meth “, “Tuk", "Speed", “Crystal")
Facts about heroin & unga
Crack/Rock, Ecstasy, and other drugs that keep you “Up”
Injection risks
Reduce injection risk
Why is it risky to use alcohol and other drugs?
Drug type activity
Where are you in having a balance in life?
Reducing alcohol & drug risks
Harm reduction strategies
The benefits of rehab
Linked factors AOD > Risky Sex > Violence
Conflict
Responses to conflict: we have a choice in the way we act and communicate
Concerns about abuse of women
Myths and truths about abuse
Rape
Myths and truths about rape
Rape and violence prevention
If in a car or taxi with a drunk or violent man
Violence prevention with boyfriend or husbands
Problem-solving steps for life
Standing your ground
Stay alert, stay powerful
Benefits of support: sister to sister: helping each other
Summary: women can become powerful: understanding intersectional risks, laws and skills for protection

Core elements of the original intervention on which the WHC is based include (1) role-playing condom use negotiation; (2) developing an individualized action plan to establish concrete risk-reduction steps, such as substance use and sexual risk behavior; (3) accessing HIV testing or making referrals to local service agencies; and (4) distributing risk-reduction materials, such as male and female condoms and lubricants. A formative phase that included focus group discussions with stakeholders and meetings with a Community Collaborative Board (CCB) was conducted to adapt and refine the intervention specifically for women living with HIV [37].

Additional slides were developed around ART, understanding viral load and the importance of “good” adherence. This resulted in a new published intervention curriculum.

### Follow-up Appointment

Participants were followed up 6 months post enrollment to take part in a follow-up appointment involving activities similar to the intake appointment for repeated measures. At this appointment, participants re-consented, participated in a risk behavior survey via computer-assisted personal interviewing, and provided a urine sample for drug screening and a breath scan via a breathalyzer for recent alcohol use. Follow-up appointments were conducted either at the study clinic or at the project field site. Participants were given R150, a certificate of study completion, and transportation at this final appointment.

### Measures

The following patient-level measures were assessed at baseline and 6-month follow-up.

#### Outcomes

ART adherence was derived from the number of participants that self-reported they received ART in the past 6 months. ART adherence was measured using a visual analogue scale (VAS) item with a line that represented adherence ranging from 0% to 100% (0% for no ART taken in the past month to 100% if ART was taken every day in the past month). Participants who reported that they were currently on ART, pointed at a percentage value on the VAS that represented their adherence in the past month. For those who had difficulty in interpreting percentages, staff asked how many days they had taken ART in the past month and their responses were then appropriately converted into percent adherence. These values were dichotomized, with reported values of 85% or greater coded as 1 (adherent) and values less than 85% coded as 0 (not adherent). Participants who were not currently on ART were coded as not adherent.

Alcohol use was assessed by self-report and breathalyzer. For self-reported alcohol use, questions about the past month frequency and amount of alcohol consumed were used to calculate/classify risk for alcohol use disorder, using the National Institute on Alcohol Abuse and Alcoholism (NIAAA) definition—for women, consuming 4 or more drinks on any given day and 7 or more drinks per week. Substance use was assessed by self-reported frequency of benzodiazepines, cocaine, methamphetamine, MDMA, marijuana, and methaqualone (Mandrax) use in the past 30 days. Additionally, substance use was assessed by urine drug screen test results.

#### Exposure

The intervention cycle, which was the order the intervention was administered, was the exposure of interest. Cycles ranged from 1 to 4.

#### Additional Covariates

Additional baseline covariates included in the propensity score (described below) all show statistically significant differences by cycle. The covariates mainly comprise self-report variables, but also two biological drug screening tests for methaqualone (Mandrax) and benzodiazepine. The remaining self-report variables span the constructs of substance use, violence, and treatment. Demographic indicators of food insecurity and main partner in the past 6 months were used. Alcohol and other drug use self-report variables include quantity of drinks on an average day and alcohol and other drug use treatment in the past 6 months. Gender-based violence indicators included ever experienced physical abuse and ever being threatened with a weapon. Finally, receiving treatment for any alcohol or other drug use in the past 6 months and awareness of CD4 count also were included. With a cluster randomized design, it is likely that the characteristics are not equivalent/balanced across the cycles (i.e., clusters).

As part of the follow-up survey, intervention satisfaction and acceptability were assessed through a modified version of the Client Satisfaction Questionnaire (CSQ-8) [38], an 8-item measure with a total score ranging from 8 to 32. Higher values indicate greater satisfaction with the intervention workshops. Descriptive analyses were conducted for this measure among participants who attended at least 1 of the 2 workshops.

### Analyses

We began by calculating descriptive statistics at baseline and follow-up for each cycle. We identified nine variables that could plausibly be related to the outcomes and that differed significantly across cycles. We included these nine variables in a multiple logistic regression analysis and saved the predicted probabilities. These predicted probabilities were entered into the outcome models as propensity scores [39]. Cycle 1 was used as the reference group to compare subsequent cycles to the first cycle, as the study design allowed for modifications and refinements after each cycle through the mixed methods design [36]. We used a generalized estimating equations (GEE) approach with an exchangeable correlation matrix to logistic regression to adjust for correlations among participants within each cycle. Analyses were conducted in SAS Enterprise Guide Version 7.15 and Stata MP/IC Version 16.

## Results

### Study Sample

**Fig. 2:**
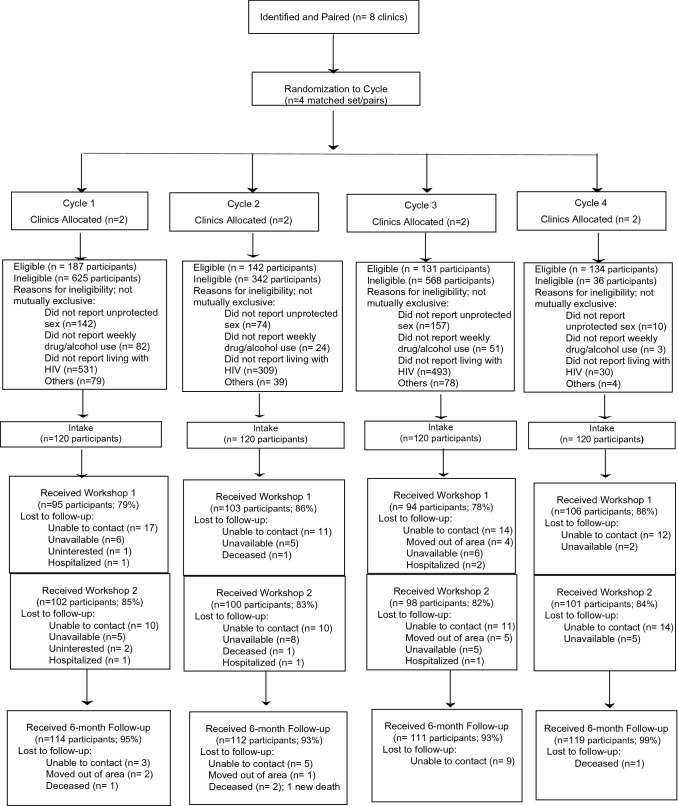
CONSORT diagram

The study’s CONSORT Diagram is presented in Fig. 2. A total of 2165 people were screened for eligibility, and 594 (27%) were eligible. Reasons for ineligibility are noted in the CONSORT Diagram. A total of 480 participants were enrolled and consented. An average of 83% of participants attended Workshop 1, ranging from 79 to 88% across the 4 cycles. An average of 84% of participants attended Workshop 2, ranging from 82 to 85% across the 4 cycles. Approximately 91% of participants received at least one workshop (not shown). Approximately 95% of participants returned for their 6-month follow-up—ranging from 93 to 99% across the 4 cycles. There were 4 non-study-related deaths over the course of the study, which were attributed to comorbidity. There was no differential attrition by cycle.

Table 2 presents the demographics of the study participants enrolled overall and across the 4 cycles (N = 480). The mean age of the study participants was 33 years (SD = 6). Most participants (93%) were Black African (not shown). A third of participants (36%) had some form of food insecurity and 46% of participants did not have running water in their home. Participants drank an average of 11 days (SD = 7) during the past month, had 14 drinks (SD = 6) per drinking day during the past month, and had an average of 11 days (SD = 7) in the past month when they drank 4 or more drinks. Among participants, 92% met the thresholds for NIAAA’s definition of risk for an alcohol use disorder. These alcohol measures differed significantly by cycle. An estimated 70% of participants reported taking ART in the past month, 72% reported taking ART in the past 6 months, and 85% reported ever taking ART—the latter was statistically significant by cycle.

**Table 2:** Demographic characteristics of Women’s Health CoOp intervention participants, overall and by cycle (N = 480)

Variable	Overall Baseline	Overall Follow-up	Cycle 1		Cycle 2		Cycle 3		Cycle 4	
% or mean (SD)	(n = 480)	(n = 456)	Baseline (n = 120)	Follow-up (n = 114)	Baseline (n = 120)	Follow-up (n = 112)	Baseline (n = 120)	Follow-up (n = 111)	Baseline (n = 120)	Follow-up (n = 119)
Age	33 (6)	33 (6)	32 (6)	33 (6)	32 (7)	33 (7)	33 (7)	34 (7)	33 (6)	34 (6)
Number of children given birth	2 (1)	–	2 (1)	–	2 (1)	–	2 (1)	–	2 (2)	–
Ever gone to bed hungry	36	–	55	–	57	–	23	–	9	–
No running water in home	46	–	48	–	48	–	40	–	48	–
Homeless	23	30	8	33	30	40	34	24	18	23
Ever been pregnant	91	–	95	–	94	–	88	–	88	–
Have main partner	86	74	88	89	83	81	94	81	78	48
Main partner in past 6 months	91	83	97	96	90	91	97	86	81	59
Less than 10^th^ grade	40	–	41	–	41	–	43	–	34	–
Given birth since HIV positive	59	–	57	–	63	–	58	–	61	–
*Substance use*										
Days drank past month	11 (7)	7 (7)	11(9)	8 (8)	11 (7)	9 (7)	11 (6)	7 (6)	13 (7)	4 (6)
Drinks on average day past month	14 (6)	10 (6)	9 (5)	8 (5)	12 (6)	12 (6)	18 (4)	7 (6)	17 (6)	12 (8)
Days 4 + drinks past month	11 (7)	7 (7)	10 (8)	8 (8)	10 (6)	9 (6)	11 (6)	6 (6)	13 (7)	5 (7)
Alcohol Use Disorder Risk	92	63	90	78	89	78	94	68	93	32
Days marijuana past month (n = 64)	17 (13)	13 (12)	15 (13)	16 (13)	17 (14)	15 (13)	16 (14)	8 (11)	22 (11)	10 (11)
AOD treatment past 6 Months	4	31	2	14	3	12	5	24	7	71
Positive benzodiazepine	3	3	6	4	3	6	2	1	1	0
Positive methaqualone (Mandrax)	9	8	3	4	15	15	10	7	6	5
*ART/ARVs*										
Know last viral load	7	12	7	18	9	16	5	8	7	5
Ever taken ART	85	–	80	–	82	–	92	–	88	–
Past 6 months taken ART	72	88	68	79	68	84	70	93	83	96
Past month taken ART	70	83	66	75	65	73	69	89	79	95
Percentage of ART taken past month	75 (40)	89 (28)	78 (38)	88 (29)	70 (41)	78 (37)	71 (44)	93 (23)	81 (35)	96 (19)
Know last CD4	40	34	48	54	49	49	33	19	32	15
*Sexual risk*										
Condom use last sex—main partner	27	44	35	51	31	46	30	41	23	38
Last sex condom use—any partner	28	58	30	50	27	48	29	50	28	81
Impaired last sex	44	31	41	32	44	37	46	39	44	18
Impaired and condomless last sex	32	15	30	18	32	17	37	23	31	5
*Gender-based violence*										
Ever physical abuse	65	–	73	–	67	–	69	–	52	–
Ever forced sex	22	–	29	–	18	–	27	–	14	–
Ever main partner physical abuse	35	–	39	–	36	–	38	–	28	–
Ever threatened with weapon	34	–	44	–	41	–	33	–	17	–

*AOD* alcohol and other drugs, *ART/ARVs* antiretroviral therapy/antiretrovirals, *NIAAA* National Institute on Alcohol Abuse and Alcoholism, *SD* standard deviation

In addition, Table 2 presents follow-up information to correspond to the baseline values. Overall, report of homelessness increased (from 23 to 30%) whereas reports of participants currently having a main partner decreased (from 86 to 74%). Overall alcohol use also reduced in average days drinking, days drinking 4 or more drinks (Mean = 7, SD = 7 for both), and average drinks per day (Mean = 10, SD = 6). Increases in ART use also were observed overall, with 88% reporting ART use in the past 6 months, 83% reporting past month ART use, and ART adherence at an average of 89% (from 75%) of ART taken in the past month.

### Logistic Regression Using Generalized Estimating Equations (GEE) Approach

GEE models examined associations between cycle and intervention outcomes of interest—alcohol use disorder risk and 85% ART adherence at follow-up. Cycle 1 was used as the reference group in the models, which also included the baseline value and a propensity score variable to control for relevant baseline differences in participant characteristics across the cycles. Compared with Cycle 1, women in Cycle 4 were significantly less likely (OR = 0.10 [95% CI 0.04, 0.24], *p* < 0.001) to report alcohol use disorder risk at 6-month follow-up. The odds were not statistically significant for alcohol use disorder risk for Cycle 2 or Cycle 3 as compared with Cycle 1 (Table 3).

**Table 3:** Women’s Health CoOp intervention effects on alcohol use disorder risk at 6-month follow-up, by cycle using generalized estimating equations (GEE) approach to logistic regression

	Logistic regression		Multiple logistic regression	
	OR (95% CI)	p value	OR (95% Cl)	p value
Alcohol use disorder risk baseline	5.31 (2.58, 10.95)	< 0.001	8.62 (3.70, 20.09)	< 0.001
Cycle		< 0.001		
Cycle 1 (reference)	‒	‒	‒	‒
Cycle 2 vs. Cycle 1	0.98 (0.52, 1.83)	0.943	1.00 (0.48, 2.06)	0.994
Cycle 3 vs. Cycle 1	0.58 (0.32, 1.06)	0.078	0.48 (0.21, 1.11)	0.085
Cycle 4 vs. Cycle 1	0.13 (0.07, 0.24)	< 0.001	0.10 (0.04, 0.24)	< 0.001
Propensity score	0.31 (0.12, 0.79)	0.014	1.06 (0.23, 4.82)	0.944

At follow-up, women in Cycle 4 were significantly more likely (OR = 4.16; 95% CI 1.05, 16.51) to report being at least 85% adherent to ART in the past 6 months compared with women in Cycle 1 (Table 4). As predicted, the likelihood of taking ART increases as women are enrolled in the later cycles and the risk of alcohol use disorder decreases.

**Table 4:** Women’s Health CoOp intervention effect on 85% or greater antiretroviral therapy (ART) adherence at 6-month follow-up, by cycle using generalized estimating equations (GEE) approach

	Logistic regression		Multiple logistic regression	
	OR (95% CI)	p value	OR (95% CI)	p value
85% ART adherence at baseline	2.02 (1.10, 3.71)	0.023	1.99 (1.08, 3.67)	0.028
Cycle		< 0.001		
Cycle 1 (reference)				
Cycle 2 vs. Cycle 1	0.51 (0.25, 1.02)	0.058	0.74 (0.32, 1.70)	0.474
Cycle 3 vs. Cycle 1	2.01 (0.86, 4.69)	0.106	2.59 (0.75, 8.93)	0.132
Cycle 4 vs. Cycle 1	3.89 (1.46, 10.41)	0.007	4.16 (1.05, 16.51)	0.043
Propensity score	2.49 (0.79, 7.82)	0.118	0.97 (0.13,7.52)	0.980

### Intervention Satisfaction

Intervention satisfaction was high among the 435 participants who participated in at least one of the intervention’s workshops, with a mean score of 30.9 (SD = 1.9) out of the possible range of 8 to 32.

## Discussion

As the importance of recognizing gender differences in intervention research increases, alcohol use in gender-related behavioral HIV prevention intervention research in the era of ART is essential to understanding intersectional risks and advancement. After numerous previous efficacy trials with the WHC, for this study, the WHC was implemented in usual care settings by trained healthcare staff and found acceptable [35]; however, these study outcomes focus on intervention effectiveness on ART adherence and alcohol reduction. While three-fourths of the participants at baseline were taking ART, our findings show that the WHC increased ART adherence and reduced alcohol use.

The goal of the WHC is to educate and offer skills and practice so that women become empowered to take better care of their health by reducing alcohol use and adhering to ART, which will lead to better overall health. This may also have a wider effect, as women are pivotal to their families as important role models for their children and also within their surrounding neighborhoods and communities.

Although intervention fidelity was high [35] and the information about the personalized action plan was presented and participants encouraged to develop their action plans, which is a core element of the intervention [40], action plans were not delivered consistently because of clinic schedules and time. However, by Cycle 3, the personalized plan component was effective and implemented more consistently at the closing of the workshop where the goals were discussed overall, with the intention of empowering women to improve their lives.

Differences between the cycles could be reflective of community population differences and the composition of clinic staff implementing the WHC intervention, in addition to the iterative process of the modified stepped-wedge, mixed methods design of learning and refinement from the previous cycle, informing how each cycle and the methods of the intervention can be improved.

One strength of the study is that there was minimal attrition at 6-month follow-up, with only 5% of participants not returning for their 6-month follow-up; and the high workshop attendance, with more than 90% of participants attending at least one workshop. We also worked with a diversity of clinic staff to train on the WHC and to have buy-in for the intervention. Also, clinic staff asked to use the intervention for other parts of their program—such as the STI information in a family group—and they wanted other people to be in their groups.

Using implementation science methods in usual care settings in South Africa is a learning process involving constraints with staff schedules and crisis management along with other staff demands. Also, space to conduct the intervention can sometimes be a challenge. Additionally, staff attitudes toward their patients can affect outcomes. These data did not address stigma toward patients based on either their HIV status or alcohol use; however, we have noted this need for future studies. We also know that women and staff were different in each cycle and clinic because they represented different communities and we attempted to discern these differences in the analyses. Finally, the follow-up period was brief at only 6 months.

Despite these limitations, this study demonstrated important reductions in the risk of alcohol use disorder given the relationship between risky alcohol use and ART adherence. South Africa remains an epicenter of alcohol use and other intersecting risks for women, who continue to bear a disproportionate burden of the HIV epidemic. To achieve the South African and UNAIDS goals for the HIV continuum of care, it is vital to address ART adherence among women living with HIV through a gender-focused lens.

## Conclusions

Led by the healthcare staff in the usual care settings, the WHC intervention was effective in reducing the risk of alcohol use disorder and increasing ART adherence among a sample of women living in economically disadvantaged communities. Consequently, continued implementation is essential. The impact of the WHC for women must continue to be disseminated back in the community through stakeholders, such as community collaborative boards, partners, and government for continued buy-in.

Determining the long-term sustainability of the WHC is a key next research question within these usual care settings. Further implementation of the WHC in additional settings or in open-air tents because of COVID-19 to minimize indoor clinic activities might be the next best solution to remain situated in clinics. Addressing stigma as a structural barrier for accessing treatment also will be an essential next step.
